# Expanding the OMOP common data model to support extracorporeal life support research

**DOI:** 10.1093/jamiaopen/ooag112

**Published:** 2026-06-22

**Authors:** Clemens Rieder, Aleh Zhuk, Ahmed Said, Andrea S Becker-Pennrich, Konstantin Dietz, Jan Bělohlávek, Jeffrey Geppert, Christopher Horvat, Ryan P Barbaro, Joseph E Tonna, Steven A Conrad, Lars Mikael Broman, Christian Reich, Clair Blacketer, Daniel Brodie, Peta M A Alexander, Dominik J Hoechter

**Affiliations:** Department of Anaesthesiology, LMU University Hospital, LMU Munich, Munich, 81377, Germany; Observational Health Data Science and Informatics, New York, NY, 10032, United States; Odysseus Data Services, an EPAM Company, Cambridge, MA, 02142, United States; Anaesthesiology and Operative Intensive Care, University Hospital Augsburg, Augsburg, 86156, Germany; Division of Pediatric Critical Care, Department of Pediatrics, and the Institute of Informatics, Data Science and Biostatistics, Washington University in St. Louis, St. Louis, MO, 63110-1093, United States; Department of Anaesthesiology, LMU University Hospital, LMU Munich, Munich, 81377, Germany; Faculty of Medicine, Institute for Medical Information Processing, Biometry and Epidemiology (IBE), Pettenkofer School of Public Health, LMU Munich, Munich, 81377, Germany; Department of Anaesthesiology, LMU University Hospital, LMU Munich, Munich, 81377, Germany; 2nd Department of Medicine-Department of Cardiovascular Medicine, First Faculty of Medicine, Charles University in Prague and General University Hospital, Prague, 12808, Czech Republic; Battelle Memorial Institute, Columbus, OH, 43201, United States; Department of Critical Care Medicine, UPMC ICU Service Center, UPMC Children’s Hospital of Pittsburgh, University of Pittsburgh, Pittsburgh, PA, 15224, United States; Division of Pediatric Critical Care Medicine and the Susan B. Meister Child Health Evaluation and Research Center, University of Michigan and C.S. Mott Children’s Hospital, Ann Arbor, MI, 48109-2800, United States; Division of Cardiothoracic Surgery, Department of Surgery, University of Utah Health, Salt Lake City, UT, 84112, United States; Department of Emergency Medicine, University of Utah Health, Salt Lake City, UT, 84112, United States; Division of Critical Care MedicineLouisiana State University Health Sciences Center Shreveport, Shreveport, LA, 71103, United States; ECMO Centre Karolinska, Astrid Lindgren Childreńs Hospital, Karolinska University Hospital, Stockholm, 17164, Sweden; Department of Physiology and Pharmacology, Karolinska Institutet, Stockholm, 17164, Sweden; Observational Health Data Science and Informatics, New York, NY, 10032, United States; Odysseus Data Services, an EPAM Company, Cambridge, MA, 02142, United States; Observational Health Data Science and Informatics, New York, NY, 10032, United States; Epidemiology Analytics, Janssen Research & Development, LLC, Raritan, NJ, 08869, United States; Department of Medicine, The Johns Hopkins University School of Medicine, Baltimore, MD, 21205, United States; Department of Cardiology, Boston Children’s Hospital, Boston, MA, 02115, United States; Department of Pediatrics, Harvard Medical School, Boston, MA, 02115, United States; Department of Anaesthesiology, LMU University Hospital, LMU Munich, Munich, 81377, Germany

**Keywords:** extracorporeal life support, critical care, routinely collected health data, open source software

## Abstract

**Objective:**

Despite the increased availability of electronic health records, open-source standardized data collection to facilitate high-resolution data during extracorporeal life support (ECLS) is lacking. This project aimed to assess the Observational Medical Outcomes Partnership Common Data Model (OMOP CDM) for interoperability to store data sufficiently generated in the context of ECLS and to develop a custom data model expansion in case the OMOP CDM proved insufficient.

**Design and Setting:**

The OMOP CDM was analyzed qualitatively by expert consensus for its capability to capture data relative to ECLS as well as the presence of fitting ECLS-related concepts. Database entries necessary to store information about primary ECLS components were compared using the OMOP CDM versus the custom data model expansion.

**Main Results:**

Analysis of data elements required to capture ECLS data within the OMOP CDM revealed a paucity of suitable concepts within the OHDSI Standardized Vocabularies, limiting capture of ECLS circuit-derived data. Custom ECLS-specific database tables and novel concepts were introduced as part of a custom expansion, the ECLS Common Data Model (ECLS CDM). The number of database entries necessary to store ECLS use cases was reduced by up to 90%. The ECLS CDM was released as an open-source project on GitHub and placed in the public domain.

**Conclusions:**

With the first iteration of the ECLS CDM, we introduce a data model to improve interoperability for data describing ECLS and elevate data quality, enabling multi-center research and quality initiatives.

## Introduction

Extracorporeal life support (ECLS) is an established support modality for patients with life-threatening cardiac and or pulmonary failure refractory to other therapies.^1^ ECLS encompasses a range of techniques, of which extracorporeal membrane oxygenation (ECMO) is the most widely used. Among the most common configurations are venoarterial (VA) and venovenous (VV) ECMO.

During ECMO, blood is drained from the patient via a venous cannula and circulated through an extracorporeal circuit where a pump drives flow and a gas exchanger adds oxygen and removes carbon dioxide. The oxygenated blood is returned via one or more distinct return cannulas. VV ECMO drains and returns blood from and to the venous system, providing respiratory support, whereas in VA ECMO, the blood is returned to the arterial system, providing cardiac and respiratory support. ECLS affects hemodynamics, coagulation, and inflammation, driven by a permanent interplay between ECLS and the patients’ physiology that can change within minutes.^2^ Different ECLS setups may be combined or modified to address various pathologies, resulting in a broad spectrum of ECLS configurations. Reconfigurations may occur multiple times during the course of therapy, further increasing the complexity of these systems. Advances in surgical methods, technology, and biocompatible materials^3^ combined with the inherent specificity of ECLS configurations and its components generate a distinct dataset (eg, rotational speed of blood pump, component properties such as cannula sizes and cannulation locations) that cannot be meaningfully reduced to a single device entry or procedure code without substantial loss of clinical information. As a consequence, a myriad of terminologies have found their way into clinical practice, often inconsistently used by clinicians and researchers in scientific reports and publications. Subsequently, a standardized ECLS nomenclature was first defined by the Extracorporeal Life Support Organization (ELSO) in the ELSO Maastricht Treaty publications.^4^^,^^5^ This standardized descriptive terminology has been the foundation for the data reporting to the ELSO Registry, but has yet to be extended to interoperable data standards. Furthermore, the relatively low prevalence of ECLS highlights the need to adopt these data standards not only for institutional, multi-center, and multinational research collaboratives, but also for their expansion to uncaptured aspects of ECLS care.

Beginning in the 1960s,^6^ efforts to standardize electronic health records (EHR) data have progressed, culminating in the development of several clinical common data models (CDM).^7–10^ CDMs facilitate the systematic analysis of disparate databases by transforming and mapping source data to clinical terminologies and storing them in a consistent data structure. Interoperability enables systems to communicate, exchange, and automatically process information.^11^ Key aspects include data harmonization on a structural level (syntactic interoperability) and standardized nomenclature (semantic interoperability), such as the Systematized Nomenclature of Medicine—Clinical Terms (SNOMED CT). CDMs are designed to support both forms of interoperability.

Previous reviews identified the Observational Medical Outcomes Partnership Common Data Model (OMOP CDM) as the most comprehensive.^12–14^ Combined with a number of powerful open-source analytics tools^15^ and an active online and offline community, the OMOP CDM is the prevalent common data model^16^ informing clinical research, a fact underlined by efforts to map other common data models to the OMOP CDM data standard.^17^^,^^18^

In this work, we evaluated the ability to capture ECLS-related data using available terminologies and data structures within the OMOP CDM. We then developed a common data model specific to ECLS to capture data generated during ECLS therapy in a format compatible with the OMOP CDM due to a lack of preexisting ECLS concepts and poor operability using only OMOP CDM standardized clinical data tables.

## Methods

### Usability analysis of the present OMOP CDM standardized clinical data tables layout for ECLS-related data capture

The OMOP CDM standardized clinical data tables (version 5.4) were evaluated through a qualitative expert consensus process to assess their effectiveness in managing ECLS-related parameters without additional database tables. Representative ECLS use cases, such as changing the cannulation configuration, clinical management, simultaneous incorporation of multiple circuits, or component exchange, were employed to assess and develop possible mapping solutions. The total number of database entries necessary to store ECLS components was compared with the number of database entries after implementing the custom data model.

### Landscape analysis of existing ECLS-related concepts and present vocabulary infrastructure

Clinical terms defined in the ELSO Maastricht Treaty and the ELSO Registry Data Definitions Document (version 04/15/2024)^19^ were cross-referenced with equivalent concepts in the Observational Health Data Sciences and Informatics (OHDSI) Standardized Vocabularies^20^ using Athena,^21^ the SNOMED CT browser (International Edition)^22^ and the Logical Observation Identifiers Names and Codes (LOINC) browser.^23^ The ELSO Maastricht Treaty, which consists of six tables, was analyzed; the first four tables covering clinical terminology were included, while the latter two, defining units of measurement and abbreviations for vascular access sites, were excluded since they contain already well-established concepts. The ELSO Registry Data Definitions Document details every mandatory and non-mandatory parameter of the ELSO Registry’s minimum dataset. For consideration, existing concepts had to be *valid* (ie, the concept is usable with the current OMOP CDM version), *standard* (ie, the concept can be used to populate specific database tables), and belong to one of the following OMOP CDM vocabulary domains, which cluster groups of similar types of medical concepts: *condition* (concepts related to state of health of the patient), *drug* (concepts related to the administration of pharmaceutical components), *device* (concepts related to the therapeutic usage of medical devices), *measurement* (concepts related to measurements), *observation* (concepts related to observations made by healthcare professionals during the hospital stay), or *procedure* (concepts related to procedures performed on the patient with the intent to treat).^24^

### Designing a common data model specific to ECLS

The OMOP CDM version 5.4 was used as a reference.^25^ Data Definition Language (DDL) files for the PostgreSQL relational database management system (version 15.2) were generated using Visual Studio Code (version 1.85.2). DBeaver Enterprise (academic license, version 23.3.0) was used to validate and test the DDLs. Following a multi-staged review process, missing concepts in OHDSI Standardized Vocabularies were created, adhering to OMOP CDM contribution guidelines.^26^ Names were chosen following SNOMED CT naming conventions to ensure consistency in wording and grammar, and were released as part of the OMOP Extension vocabulary.

### Mapping of an deidentified dataset to the OMOP CDM and ECLS CDM

To demonstrate the feasibility of the ECLS CDM extension, we analyzed a deidentified dataset of 326 ECLS runs over a 6-year period from Washington University in St. Louis and outlined a mapping process, along with guidelines for mapping ELSO Registry data to the OMOP CDM and ECLS CDM. The dataset included 175 fields (columns) across nine tables. Standardized terminologies in the source data, including ICD-10 and CPT-4 codes, were mapped to standard concepts using OHDSI standardized vocabularies. Target domains determined the appropriate OMOP CDM and ECLS CDM destination fields. Fields without standard coding, such as laboratory data, ECLS-specific parameters, or event-based annotations, required manual semantic mapping. One of the main challenges was mapping time-relative data. The ELSO Registry frequently uses time offsets relative to index events (eg, cannulation, decannulation), whereas OMOP CDM requires absolute dates and datetimes. Where anchor dates were available, we calculated absolute timestamps. When this was not possible, dates were imputed based on available context.

### Stakeholder engagement and composition of the study team

To develop a comprehensive data model that equally meets the needs of ECLS clinicians and data and health research communities, ELSO’s board of directors and OHDSI’s management were approached to form a multidisciplinary study team. Initial concepts and a preliminary draft of the data model were presented by authors DH and CR during official meetings between April 2023 and August 2023. The study team was composed of experts with various backgrounds in clinical and non-clinical sciences, representing expertise in ECMO, electronic health records, public health, and data science, as well as leadership in the professional societies relevant to the data model. The core team of four physicians (DJH, CR, AS, AZ) with expertise in medical informatics as well as adult, pediatric, and neonatal ECLS therapy developed the concepts and relationships in regular meetings (monthly on average) over the course of 18 months. Conflicts were thoroughly discussed until a unanimous solution was reached among these experts. Preliminary results were presented intermittently to ECLS (JB, DB, JT, RPB, SC, LMB, PMAA) and medical informatics (CH, JG, SC, ASBP, KD) experts. Any criticism raised was revisited and debated until no objections remained. Throughout the design process, the core team collaborated regularly with OMOP CDM data scientists (CB, ChR) to ensure adherence to their stringent guidelines and quality standards. The resulting work was reviewed by the OHDSI vocabulary team. Likewise, results were presented to the Board of Directors of ELSO for approval.

### Ethical considerations

As an in-silico investigation, no human participants were involved. Analysis of the deidentified dataset was determined not to be human subject research by the Washington University in St. Louis Institutional Review Board.

## Results

### Usability analysis of the OMOP CDM standardized clinical data tables for ECLS-related data management

**Figure 1.**
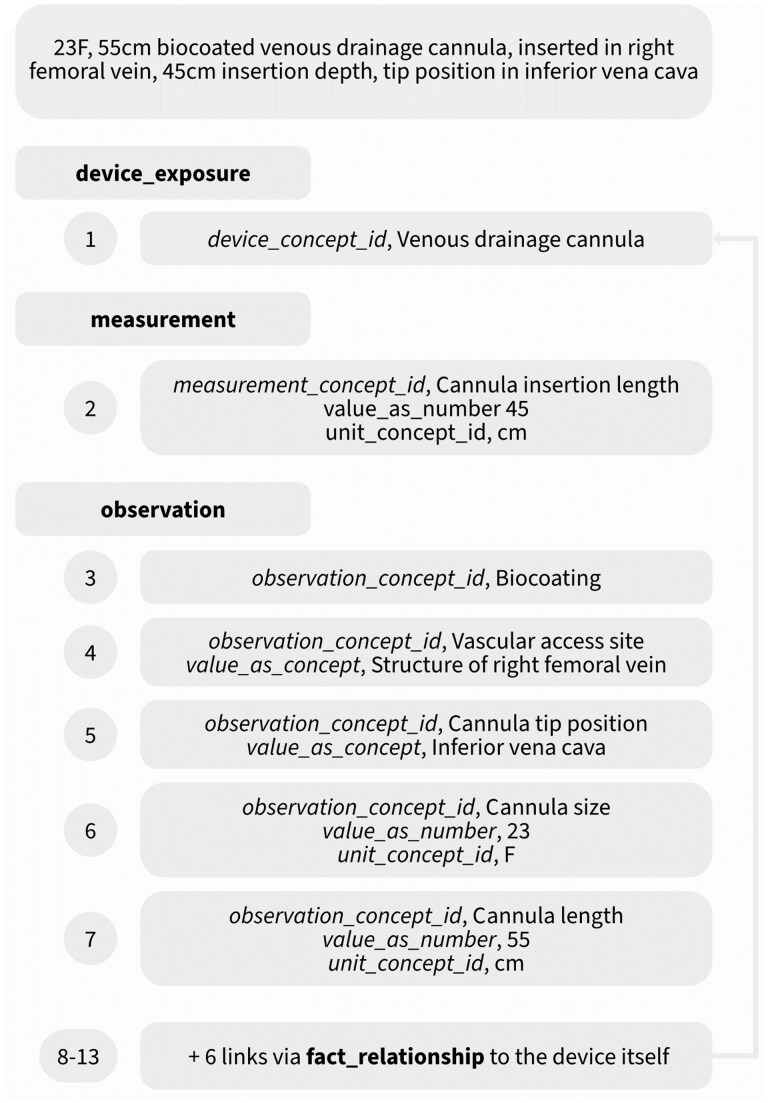
Mapping a cannula in the existing OMOP CDM.To be correctly reflected in the database, a single cannula requires up to 13 entries (depending on the concepts and disregarding the mandatory link to the person table) in different tables. These entries are connected to the main entry in device_exposure using the fact_relationship table. OMOP CDM database tables are in bold; numbers indicate the necessary entries.

Preliminary assessments revealed that, while it was feasible to map some ECLS data using only OMOP CDM standardized tables, there were significant limitations: usability was substantially reduced, leading to increased complexity and impractical mapping guidelines. A major constraint was that the data generated by the components of an ECLS circuit required representation across multiple tables within the OMOP CDM, necessitating linkage via the fact_relationship table. This additionally amplified the complexity of using Structured Query Language (SQL) for data analysis. Figure 1 illustrates an example of mapping a single ECLS component using only the clinical data tables of the OMOP CDM. Furthermore, using the fact_relationship table resulted in the loss of temporal information, as it is designed to develop connections between tables but lacks the capacity to capture timestamps, thus prohibiting the determination of duration between these connections.

### Concept analysis of the ELSO Maastricht Treaty and the ELSO registry

Of the 75 concepts in the ELSO Maastricht Treaty, 19 (25%) were directly mappable to existing valid and standard concepts within the OMOP CDM. One concept (1%) could be represented via post-coordination, allowing a logical combination of concepts instead of one equivalent or semantic synonym. However, 55 (74%) concepts could not be matched without loss of semantic fidelity. Among those, 28 (51%) concepts were designed as there were no equivalent concepts, while 27 (49%) were excluded from the design process, such as historic, duplicate, or irrelevant concepts with limited applicability for current ECLS therapy.

Four hundred and thirty-eight concepts from the ELSO Registry Data Definitions Document were analyzed, excluding those from the Current Procedural Terminology, version 4 (CPT-4) or ICD (version 10, including modifications), as they are already part of the OMOP CDM vocabulary set. Fifty-one concepts (12%) required the development of new concepts, while 387 (88%) were excluded from the design process as equivalent concepts or table attributes were already available within the OHDSI Standardized Vocabularies. Twenty-one of the 51 newly introduced as well as 108 of the 387 excluded concepts are mandatory data elements of the ELSO Data Definitions Document. A comprehensive list of these concepts can be found in the supplementary material.

### Common data model design

**Figure 2.**
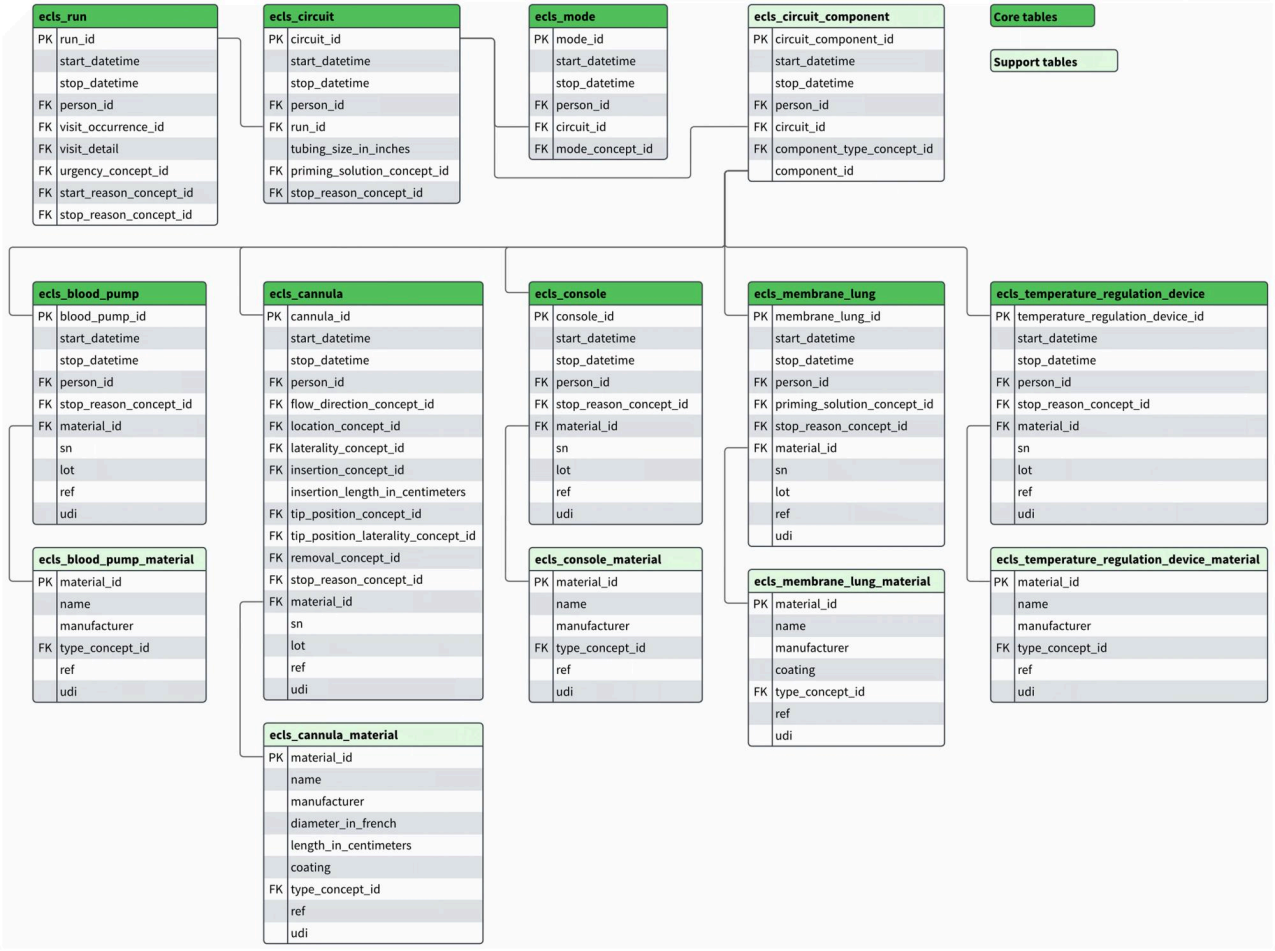
A simplified entity-relationship diagram of the ECLS CDM. The core tables are depicted in dark blue, and the support tables are in light blue. Every core table is linked to the person table of the OMOP CDM. FK denotes a foreign key, whereas PK identifies the primary key column (ie, the unique identifier of each entry), lines indicate the foreign key relationship. Columns ending in concept_id are linked to the OMOP CDM *concept* table.

**Figure 3.**
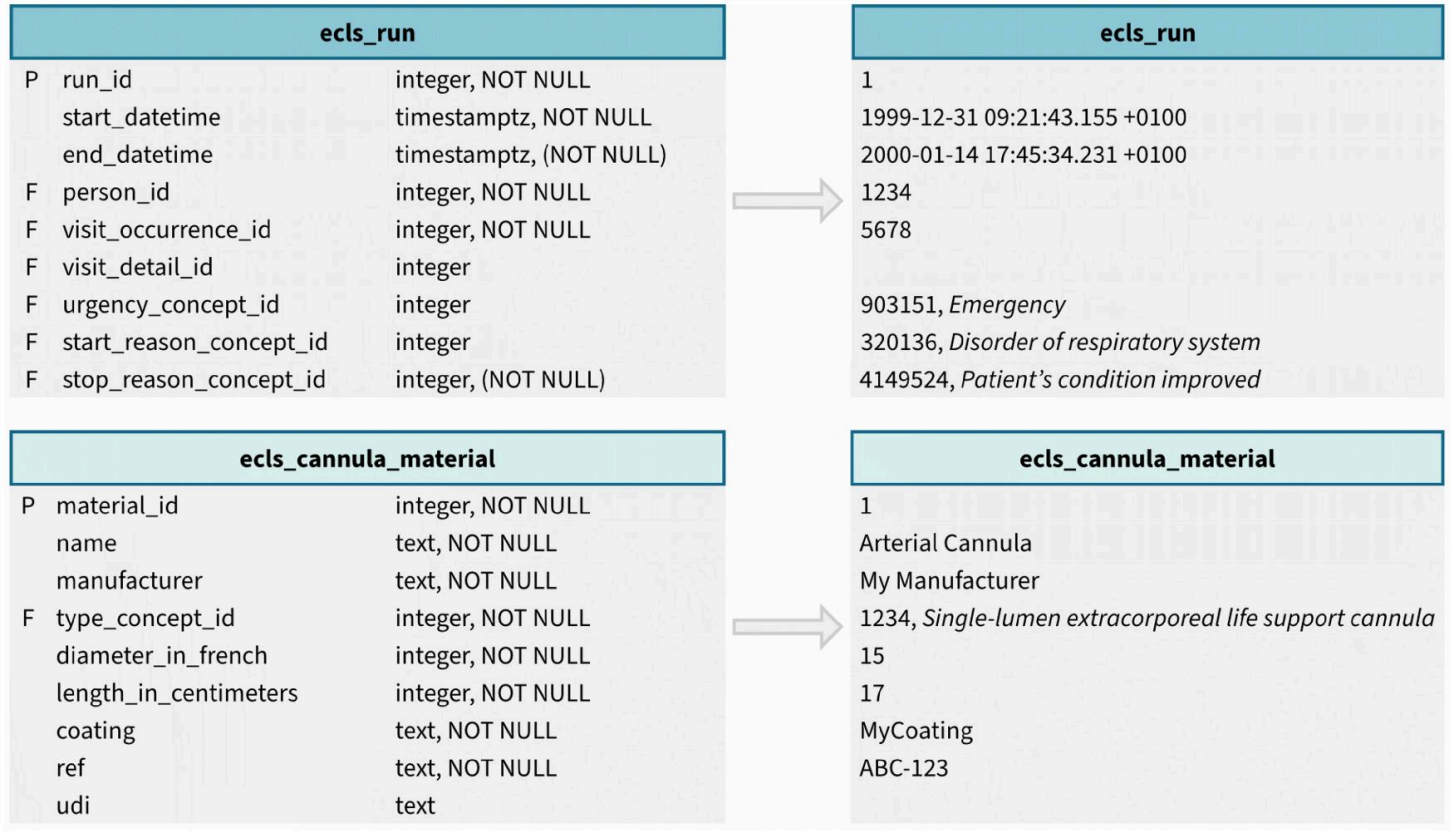
Examples of the table structure for the core table ecls_run and the support table ecls_cannula_material with example entries: on the right-hand side, a default data tuple is depicted: it displays the first ECLS run of this patient during that particular hospital stay, which was emergently started for respiratory insufficiency. (NOT NULL) describes fields that can be empty at the beginning of the data capture but must be filled in at the end of ECLS therapy. F denotes foreign key relationships, and P identifies the primary key column.

**Figure 4.**
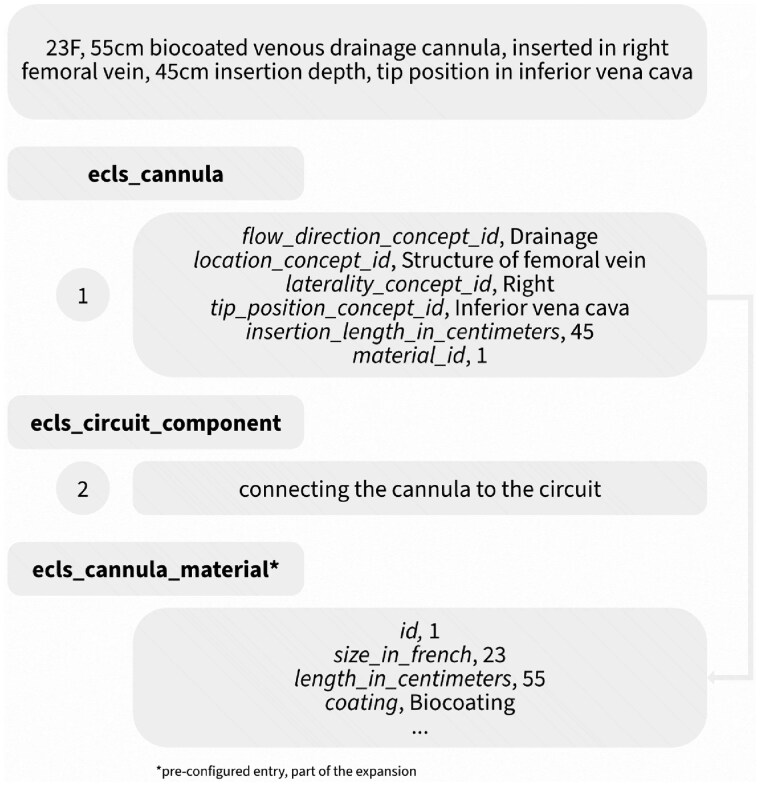
Depiction of mapping a cannula with the ECLS CDM expansion: Whereas up to 13 entries were necessary using the mainly pre-coordinated OMOP CDM, the ECLS CDM will store the same amount of information with significantly fewer entries.

Due to overall usability concerns and the absence of semantic models, a custom OMOP CDM expansion termed the Extracorporeal Life Support Common Data Model (ECLS CDM) was developed. ECLS CDM database tables were designed following best practices, including defining adequate primary key constraints to identify data tuples correctly and minimizing the number of nullable attributes. PostgreSQL was selected as the primary open-source relational database management system because of its increasing adoption over the last decade^27^ and feature advantages over the more widely used open-source alternative MySQL.^28^^,^^29^ ECLS CDM tables were logically divided into core and support tables. Core tables are directly linked to the OMOP CDM standardized clinical data tables via a foreign key relationship to the person table. In contrast, support tables are exclusively linked to core tables, providing more detailed information about the components and establishing many-to-many relationships between components. A dedicated table was introduced for each major ECLS circuit component to capture component-specific data (Table 1). Figure 2 provides a detailed description of the database table layout, and Figure 3 shows an in-depth view of two representative ECLS CDM tables, with other expansion tables following a similar design. The ECLS CDM table design significantly reduced complexity, as shown in Figure 4, which represents the same information about the cannulation strategy as Figure 1 but in a more streamlined format. A reduction in the number of necessary database entries was observed for all major ECLS components, with relative reductions ranging from 50% for the *ecls_mode* table (1 entry instead of 2) to 90% for the *ecls_cannula* table (3 entries instead of 29), averaging a 68% reduction overall, when using the ECLS CDM to store core concepts instead of the OMOP CDM without the expansion tables. The exact counts of database entries required are shown in Figure S1.

**Table 1.** List of tables of the ECLS CDM expansion: a dedicated table for each major extracorporeal life support circuit component was introduced to capture data specific to the component.

Table name	Stores information about the…
Core tables	
ecls_run	…nature of the run itself.
ecls_circuit	…circuit in use. A run can consist of multiple circuits.
ecls_mode	…mode (configuration) of the circuit.
ecls_blood_pump	…blood pump, transporting blood through the ECLS circuit.
ecls_cannula	…cannula(s), used to drain and return blood from the patient.
ecls_console	…console of the circuit, necessary for the correct settings of the circuit.
ecls_membrane_lung	…membrane lung(s) responsible for blood oxygenation and decarboxylation.
ecls_temperature_regulation_device	…temperature regulation device warming the blood flowing through the extracorporeal circuit.
Support tables	
ecls_blood_pump_material	…material used for the blood pump.
ecls_cannula_material	…material used for the cannula(s).
ecls_console_material	…material used for the console.
ecls_membrane_lung_material	…material used for the membrane lung(s).
ecls_temperature_regulation_device_material	…material used for the extracorporeal temperature regulation device.
ecls_circuit_component	…the many-to-many relationships connecting the components to a circuit, enabling generic configurations.

### Concept development

**Figure 5.**
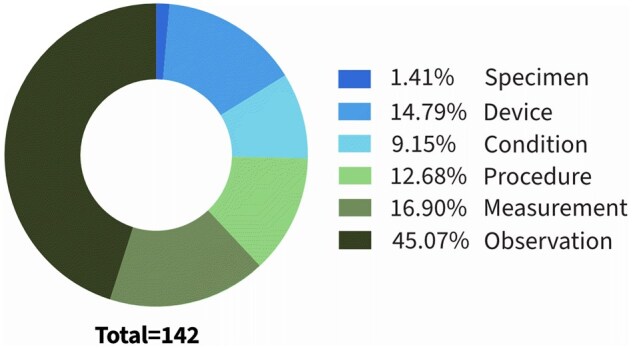
Domain distribution of novel concepts as part of the first release of the ECLS CDM.

As a direct result of the prior analyses, 134 ECLS-specific concepts were designed and incorporated in the OMOP CDM in the February 2024 release, followed by 8 concepts released during the August 2024 vocabulary release.^30^ The detailed list of novel concepts is available in the online supplementary material (supp_concepts.csv). Almost half of the new concepts were included in the observation domain, followed by measurement and procedure domains (Figure 5). The new concepts were released as part of the OMOP Extension vocabulary and integrated into the existing hierarchy via *is_a* relationships to either newly introduced parent concepts belonging to the OMOP Extension vocabulary or preexisting SNOMED CT concepts. Afterward, clinical synonyms were mapped to one fully specified name to enhance their acceptance and simplify search queries. Candidate concepts deemed outdated, mappable via post-coordination, or unlikely to be implemented by the OMOP CDM users were excluded after expert consensus. The ELSO Registry addenda containing concepts specific to certain ECLS use cases were not analyzed for the initial release of the data model.

### Mapping results for deidentified ELSO registry data

One hundred and ten columns could be mapped, whereas 65 columns were excluded from the test-wise ETL (Extract, Transform, Load) process. The excluded columns could not be mapped directly to the CDM for the following reasons: missing semantic context or time-related information of the data, duplicative information, calculated values based on other columns that could be normalized, or missing field information in the current ELSO Registry Data Definition document. Imputed values were used in the majority of temporal fields, since the deidentified dataset provided only absolute timestamps for two columns.

### Rollout of the common data model

All data definitions and concepts related to the ECLS CDM expansion were made publicly available on the respective ECLS CDM GitHub repository under the CC0-1.0 license.^31^ The expansion adhered to Semantic Versioning principles,^32^ and changes were tracked via the changelog file following the rules of Keep a Changelog.^33^ Markdown files were processed via the Python extension mkdocs (version 1.5.3) to create the online documentation using Python version 3.14.

## Discussion

In this work, we demonstrated the inadequacy of currently available data interoperability standards to capture data associated with ECLS use. Current data capture in clinical and administrative registries is often constrained by the need for manual collection, which limits the data elements included and, particularly, the ability to capture repeat measurements. In contrast, common data models, such as the OMOP CDM, are designed for longitudinal data capture and support federated analyses, enhancing data governance and security via decentralized data processing.

Our landscape analysis of core concepts from the ELSO Maastricht Treaty and the ELSO Registry Data Definitions Document highlighted the generic nature of existing OMOP CDM data tables and the absence of usable concepts in international terminologies for extracorporeal life support. Semantic fidelity is often compromised when researchers are limited to generic concepts. For example, the procedure of *decannulation* and the adverse event of an *accidental decannulation* (two terms from the Maastricht Treaty) can only be represented by one SNOMED CT concept, *Vascular cannula removal* (SNOMED CT ID: 233553003), losing any information about the nature of the removal itself. While it is possible to store ECLS-related data using only OMOP CDM standardized clinical data tables, this approach proved inefficient. Although low-level concepts can be added to the custom OMOP Extension vocabulary via community contributions, they would need to be represented across multiple database tables, leading to the fragmentation of clinically coherent information–especially since no official guidance exists for representing ECLS data using the OMOP CDM. Additionally, given that major medical terminologies typically combine pre- and post-coordinated concepts^34^ and that OMOP CDM provides only limited support for post-coordination to adequately represent ECLS therapy, a large number of new concepts would be required.

These limitations are particularly observable when considering cannula-related analyses. While core attributes such as patient linkage have clear targets, ECLS-relevant parameters, including flow direction, insertion site and laterality, cannula size, or removal characteristics, might be captured inconsistently, necessitating the use of user-formulated concept sets and complex query logic. Moreover, several key attributes, including insertion length, tip position projection onto an anatomic structure, tip position laterality, or material coating, can not be represented in the OMOP CDM (Table 2). Clinically relevant analyses such as stratification by cannula tip position or evaluation of insertion characteristics are therefore not feasible using either the source registry data or the OMOP CDM.

**Table 2.** Representation of cannula-related data elements in the ELSO Registry, OMOP CDM, and ECLS CDM.

Database column	Data present in the ELSO Registry	Data points representable in OMOP CDM	Corresponding OMOP CDM table	Data points representable using the ECLS CDM	Corresponding ECLS CDM table
cannula_id	n/a	Present	device_exposure	Present	ecls_cannula
start_datetime	Present	Present	device_exposure	Present	ecls_cannula
stop_datetime	Present	Present	device_exposure	Present	ecls_cannula
person_id	Present	Present	device_exposure	Present	ecls_cannula
flow_direction_concept_id	Present	Present, no standardized mapping	Observation	Present	ecls_cannula
location_concept_id	Present	Present, no standardized mapping	Observation	Present	ecls_cannula
laterality_concept_id	Present	Present, no standardized mapping	Observation	Present	ecls_cannula
insertion_concept	Partial	Present, no standardized mapping	Observation	Present	ecls_cannula
insertion_length_in_centimeters	Missing	Not present	N/A	Present	ecls_cannula
tip_position_concept_id	Missing	Not present	N/A	Present	ecls_cannula
tip_position_laterality_concept_id	Missing	Not present	N/A	Present	ecls_cannula
removal_concept_id	Present	Present, no standardized mapping	Observation	Present	ecls_cannula
stop_reason_concept_id	Present	Not present	N/A	Present	ecls_cannula
serial number	Missing	Present	Observation	Present	ecls_cannula
lot number	Missing	Not present	N/A	Present	ecls_cannula
reference number	Partial	Not present	N/A	Present	ecls_cannula

The table compares all attributes with respect to their presence, standardization, and structural implementation across the source dataset and both data models.

As a consequence, we developed the ECLS CDM expansion, introducing customized database tables and novel concepts specific to extracorporeal life support. This approach provides several key advantages. It supports real-time data capture, enabling high-resolution representation of ECLS-related parameters. The clear data structure enables more explicit querying, improving accessibility for clinical and research applications, and preserves temporal relationships between individual ECLS components and configurations, avoiding information loss when combinations change over time. Furthermore, the ECLS CDM supports a standardized ETL endpoint for mapping ELSO Registry data to the OMOP CDM. Users might find it easier to transform data into a well-defined, encapsulated data model that serves a specific purpose, rather than develop user- or center-specific mapping strategies for high-level OMOP CDM standardized clinical data tables. However, successful mapping is inherently tied to the quality of the source data, even when the target structure fully accounts for its specific nature. Registry data is often aggregated and deidentified to preserve patient privacy, rendering high-resolution data collection or conversion impossible. In such cases, original or Admission-Discharge-Transfer (ADT) datasets are necessary to integrate the different systems and avoid loss of semantic information. Depending on the quality of the source data, researchers might find it of little value to put effort into retrospectively transforming (already aggregated) data into longitudinal data models, unless with the purpose of interoperability or synthesis with other clinical information models in mind. As a result, the ECLS CDM is best suited as a primary data-capture model rather than a retrospective transformation target.

Nevertheless, several limitations must be acknowledged. The development of the ECLS CDM is primarily based on the ELSO Registry, which serves as the gold standard for ECLS data collection. However, concepts specific to ECLS scenarios, such as trauma, cardiac procedures, or extracorporeal cardiopulmonary resuscitation (ECPR), all of which are referred to as “Addenda” in the ELSO Registry, were not included in the initial release. Additionally, other EHR architectures, registry formats, and international coding vocabularies may limit the generalizability of the ECLS CDM. It is only partially compatible with standard OHDSI tools, such as the Hades analytic package^35^ and Atlas,^36^ limited by the fact that the new tables of the ECLS CDM are not directly accessible via these tools. For complex ECLS-specific analyses, custom SQL queries are thus necessary. For simpler use cases, additional concepts were introduced for use with the OMOP CDM standardized clinical data tables when high-level data capture suffices.

Our initial version prioritized the database layout, the implementation of a baseline set of concepts, and guidelines for both ad hoc and retrospective data collection. The local implementation of common data models, however, requires access to digitalization and on-site expertise. The ECLS CDM itself introduces additional complexity due to intricate logic and mandatory manual user input, possibly hindering swift adoption across the community. Future work may explore interface-oriented approaches to streamline user interaction with the ECLS CDM. To address these challenges, continued collaboration between the study group, ELSO, and OHDSI will be essential. Subsequent releases of the ECLS CDM will incorporate these addenda and expand compatibility with the broader OHDSI analytic toolchain.

## Conclusion

In this work, we present the first iteration of the Extracorporeal Life Support Common Data Model (ECLS CDM), an open-source expansion to the Observational Medical Outcomes Partnership Common Data Model (OMOP CDM), specifically designed to enable the standardized capture and processing of data collected in the context of extracorporeal life support. We aim to continuously update and develop the model to fit the ECLS community’s needs for a comprehensive, standardized solution to ECLS-related data management. This work is another step toward improving the overall quality of care and patient outcomes by facilitating future research and quality measurement using a common data language stored in a unified data model.

## Supplementary Material

ooag112_Supplementary_Data

## Data Availability

The data underlying this article will be shared on reasonable request to the corresponding author.
