# Supplementary figures and images for: Expanding the OMOP common data model to support extracorporeal life support research
Source: JAMIA Open. 2026 Jun 22;9(3):ooag112. doi: 10.1093/jamiaopen/ooag112 (PMC13284988; doi:10.1093/jamiaopen/ooag112)

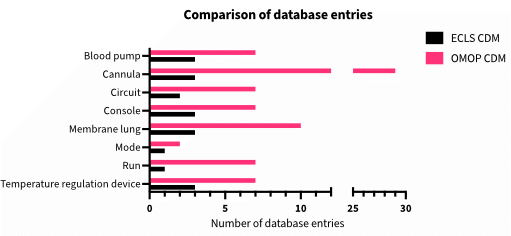

Supplement: ooag112_Supplementary_Data [file ooag112_supplementary_data.zip › Figure S1.png]
